# Survival of Patients with Hepatocellular Carcinoma in Renal Insufficiency: Prognostic Role of Albumin-Bilirubin Grade

**DOI:** 10.3390/cancers12051130

**Published:** 2020-04-30

**Authors:** Shu-Yein Ho, Chia-Yang Hsu, Po-Hong Liu, Chih-Chieh Ko, Yi-Hsiang Huang, Chien-Wei Su, Rheun-Chuan Lee, Ming-Chih Hou, Teh-Ia Huo

**Affiliations:** 1 Department of Medicine, Taipei Veterans General Hospital, Taipei 11217, Taiwan; zawzaw222@gmail.com (S.-Y.H.); ccko2@vghtpe.gov.tw (C.-C.K.); yhhuang@vghtpe.gov.tw (Y.-H.H.); cwsu2@vghtpe.gov.tw (C.-W.S.); mchou@vghtpe.gov.tw (M.-C.H.); 2 Faculty of Medicine, National Yang-Ming University School of Medicine, Taipei 11217, Taiwan; chiayanghsu2@gmail.com (C.-Y.H.); stuartliu@gmail.com (P.-H.L.); rclee@vghtpe.gov.tw (R.-C.L.); 3 Division of Gastroenterology and Hepatology, University of Michigan, Ann Arbor, MI 48109, USA; 4 Department of Internal Medicine, University of Texas Southwestern Medical Center, Dallas, TX 75390, USA; 5 Institute of Clinical Medicine, National Yang-Ming University School of Medicine, Taipei 11217, Taiwan; 6 Department of Radiology, Taipei Veterans General Hospital, Taipei 11217, Taiwan; 7 Department of Medical Research, Taipei Veterans General Hospital, Taipei 11217, Taiwan; 8 Institute of Pharmacology, National Yang-Ming University School of Medicine, Taipei 11217, Taiwan

**Keywords:** renal insufficiency, hepatocellular carcinoma, ALBI grade

## Abstract

Renal insufficiency (RI) is commonly seen in patients with hepatocellular carcinoma (HCC). The prognostic role of albumin-bilirubin (ALBI) grade in this special setting is unclear. We aimed to investigate the role of ALBI grade associated with the impact of RI on HCC. A prospective cohort of 3690 HCC patients between 2002 and 2016 were retrospectively analyzed. The Kaplan–Meier method and multivariate Cox proportional hazards model were used to determine survival and independent prognostic predictors. Of all patients, RI was an independent predictor associated with decreased survival. In multivariate Cox analysis for patients with RI, α-fetoprotein level ≥20 ng/mL, tumor size >3 cm, vascular invasion, distant metastasis, presence of ascites, performance status 1–2, performance status 3–4, and ALBI grade 2 and grade 3 were independent predictors of decreased survival (all *p* < 0.05). In subgroup analysis of patients with RI undergoing curative and non-curative treatments, the ALBI grade remained a significant prognostic predictor associated with decreased survival (*p* < 0.001). In summary, HCC patients with RI have decreased survival compared to those without RI. The ALBI grade can discriminate the survival in patients with RI independent of treatment strategy and is a feasible prognostic tool in this special patient population.

## 1. Introduction

Hepatocellular carcinoma (HCC) is the fourth leading cause of cancer mortality globally, accounting for nearly 700,000 deaths/year [1]. In Taiwan, it is the first and second leading cause of cancer-associated death in males and females, respectively [2]. Patients with HCC often have coexisting liver cirrhosis due to chronic hepatitis B or C, alcoholism, or nonalcoholic fatty liver disease [3,4]. Notably, cirrhosis is known as a predisposing factor for renal insufficiency (RI). The mechanisms are likely due to reduced systemic vascular resistance, splanchnic arterial vasodilation, decreased effective blood volume, and activation of vasoconstrictor system that cause renal vasoconstriction and hypoperfusion [5].

The presence of RI is usually defined as reduced estimated glomerular filtration rate (eGFR) below 60 mL/min/1.73 m^2^ [6]. Up to 25% of HCC patients may have RI at the time of diagnosis [7]. A major clinical impact is that earlier small series studies showed that RI was associated with poor outcome in HCC patients undergoing surgical resection, local ablation therapy, and transarterial chemoembolization (TACE) [8,9,10].

The severity of liver dysfunction plays a crucial role in the management of HCC [4]. Traditionally, the Child–Turcotte–Pugh (CTP) classification is used to evaluate the severity of liver damage. The CTP classification has its drawbacks because of subjective variables such as the degree of ascites and encephalopathy and arbitrarily defined cut-off points for objective parameters [11]. The model for end-stage liver disease (MELD) is based on serum creatinine, bilirubin, and international normalized ratio of prothrombin time to assess liver functional reserve. However, the MELD score was primarily designed for prioritization of cirrhotic patients awaiting liver transplantation unrelated to HCC [12]. More recently, the albumin-bilirubin (ALBI) grade was introduced as a marker of liver functional reserve in HCC [13]. It is a more objective measurement of liver dysfunction in HCC and has been validated by several independent study groups [11,13,14,15,16]. About 70%–90% of HCC develop on the background of chronic liver disease or cirrhosis with variable degrees of liver injury [17,18]. However, the severity of liver injury and its association with RI in outcome prediction for HCC is unclear; no study to date has investigated the role of ALBI grade in the setting of HCC patients with RI. In this study, we aim to investigate the survival of patients with HCC in RI associated with ALBI grade in a large prospectively followed-up cohort.

## 2. Methods

### 2.1. Patients

Between 2002 and 2016, 3690 newly diagnosed HCC patients who were admitted to Taipei Veterans General Hospital, Taiwan, were prospectively identified and retrospectively analyzed. The baseline information including patient demographics, etiology of chronic liver disease, serum biochemistry, severity of liver cirrhosis, tumor burden, performance status, and cancer stage were collected at the time of diagnosis. Patient survival was observed every 3–4 months until drop-out from the follow-up program or death at the end of December 2018. The study was approved by the Institutional Review Broad (IRB) of Taipei Veterans General Hospital (IRB approval number: 2020-02-004CC) and complied with the standards of the Declaration of Helsinki and current ethical guidelines. Waiver of consent was obtained, and patient records were anonymized and de-identified prior to analysis.

### 2.2. Diagnosis and Definitions

The diagnosis of HCC was based on typical enhancement pattern in the arterial phase and delayed wash-out in the portal-venous phase of contrast-enhanced computed tomography (CT) scan or magnetic resonance imaging (MRI), or histologically confirmed if the imaging finding was not typical [19]. Patients who were seropositive for hepatitis B surface antigen (HBsAg), seronegative for antibody for HCV (anti-HCV), and without alcoholism were defined as HBV-related HCC. HCV-related HCC was defined as patients seropositive for anti-HCV, seronegative for HBsAg and no history of alcoholism. The performance status was evaluated by using the Eastern Cooperative Oncology Group Performance scale ranging from 0 (asymptomatic) to 4 (confined to bed) [20]. The eGFR was calculated by using the modification of diet in renal disease (MDRD) equation: eGFR (mL/min/1.73 m^2^) = 186 × (creatinine (mg/dL))^−1.154^ × (age (years))^−0.203^ × 0.742 (if female) [21]. All patients in this study were ethnically Chinese. The serum creatinine level used in the MDRD formula was the level recorded at the time of diagnosis.

The ALBI score was calculated as the following equation = 0.66 × log_10_ bilirubin (μmol/L) − 0.085 × albumin (g/L). ALBI grade was classified as grade 1 (score ≤ −2.60), grade 2 (score > −2.60 and ≤ −1.39), and grade 3 (score > −1.39) [13,15,16]. Total tumor volume (TTV) was calculated by using the following equation 4/3 × 3.14 × (maximum radius of the tumor nodule in cm)^3^ as previously described [22]. Cancer stage was determined based on the currently recommended Barcelona Clinic Liver Cancer (BCLC) system.

### 2.3. Treatment

Patients were reviewed at our multidisciplinary HCC team for diagnosis and treatment recommendation. The criteria for surgical resection and surgical techniques were described previously in detail [23]. Briefly, resection was performed in patients with tumor(s) involving no more than three Couinaud segments with no evidence of main portal vein trunk involvement. Radiofrequency ablation was indicated in patients who had small HCC but unsuitable for surgical resection [24]. The criteria for transarterial chemoembolization (TACE) were unresectable HCC with adequate liver functional reserve and no main portal vein thrombosis [25]. Patients with advanced stage HCC were treated with targeted therapy [4]. Best supportive care was indicated for patients with terminal stage HCC [3]. In this study, surgical resection, percutaneous ablation therapy, and liver transplantation were defined as curative treatment; TACE, targeted therapy, systemic chemotherapy and best supportive care were collectively classified as non-curative treatment.

### 2.4. Statistics

Continuous variables were compared by the Mann–Whitney U test. The two-tailed Fisher exact test and Chi-squared test were applied to compare categorical variables between two groups. The survival distributions of patients were analyzed by the Kaplan–Meier method with a log-rank test. To determine the factors associated with survival, univariate analysis was performed for age, sex, etiology of chronic hepatitis, laboratory data, severity of liver cirrhosis, performance status, and cancer stage. Prognostic factors that were significantly linked with survival in univariate analysis were introduced into the multivariate Cox proportional hazards model to identify their adjusted hazard ratio (HR) and 95% confidence intervals (CI). A *p*-value less than 0.05 was considered statistically significant. All statistical analyses were conducted by using IBM SPSS Statistics for Windows, Version 21.0 (IBM Corp., Armonk, NY, USA).

## 3. Results

### 3.1. Patient Characteristics

**Figure 1:**
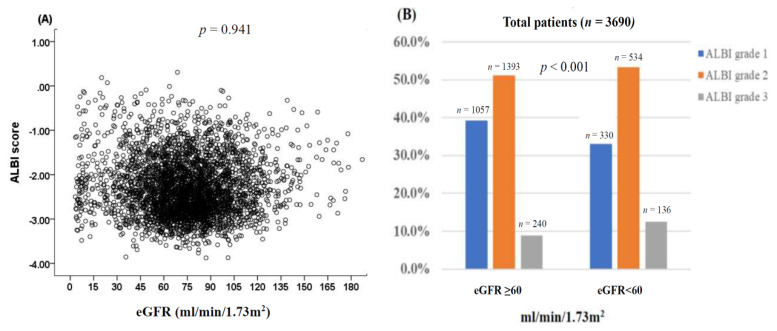
(**A**) The correlation of estimated glomerular filtration rate (eGFR) and albumin-bilirubin (ALBI) score. There was no significant correlation between eGFR and ALBI score (*p* = 0.941). (**B**) Distribution of ALBI grade was stratified by the eGFR at a cutoff of 60 mL/min/1.73 m^2^. The distribution of patients based on ALBI grade was 1057 patients (ALBI grade 1), 1393 patients (ALBI grade 2), and 240 patients (ALBI grade 3) in eGFR ≥ 60 mL/min/1.73 m^2^ and 330 patients (ALBI grade 1), 534 patients (ALBI grade), and 136 patients (ALBI grade 3) in GFR < 60 mL/min/1.73 m^2^. Patients with ALBI grade 2 or 3 were more often associated with lower eGFR (*p* < 0.001).

The baseline characteristics of patients with and without RI are shown in Table 1. The mean age of the entire cohort was 65 years, and the majority of patients were male (76%). About 40% of patients had HBV infection and 26% of patients had diabetes mellitus (DM). Most (73%) patients were CTP classification A; 38% and 52% of patients were ALBI grade 1 and grade 2, respectively. In addition, 64% of patients had a single tumor with a mean tumor size of about 6 cm, and 24% of patients had vascular invasion and 10% of patients had distant metastasis. About 81% of patients had performance status 0 to 1 at disease presentation. Further, 32% of patients were BCLC stage 0 to A, and 47% of patients underwent curative treatments such as surgical resection or percutaneous ablation. There was no significant correlation between ALBI score and eGFR (Figure 1A, trend analysis *p* = 0.941). However, ALBI grade 1 patients more often had higher eGFR, whereas grade 3 patients more often had lower eGFR (Figure 1B, *p* < 0.001).

The prevalence of RI in the entire cohort was 27%. Patients with RI were older (*p* < 0.001) with female predominance (*p* = 0.008) compared with patients without RI. Patients with RI more often had a lower serum albumin level (*p* < 0.001), higher serum bilirubin level (*p* = 0.003), lower serum α-fetoprotein (AFP) level (*p* = 0.004), larger tumor size (*p* = 0.026), more advanced stage cirrhosis (*p* < 0.001), higher prevalence of DM (*p* < 0.001), poor performance status (*p* < 0.001), more advanced BCLC stage (*p* < 0.001), and lower rate of curative resection (*p* < 0.001) in comparison to patients without RI.

### 3.2. Survival Prognostic Predictors

**Figure 2:**
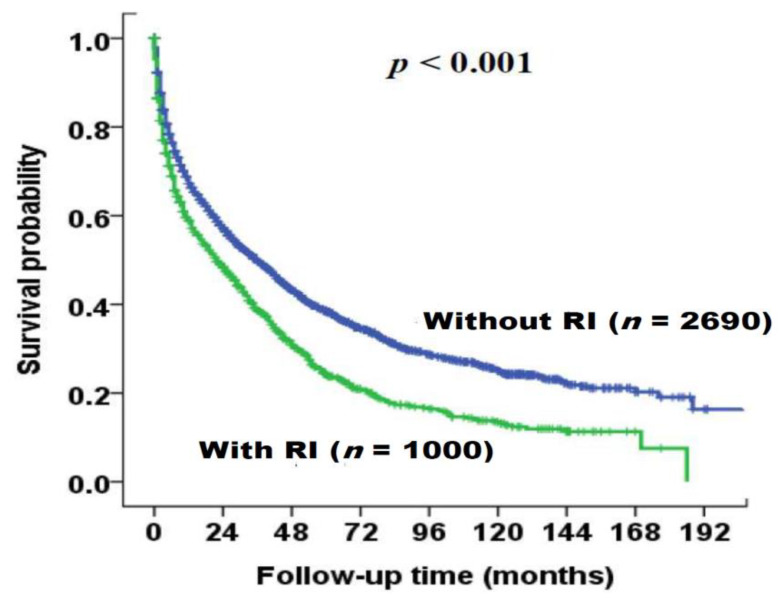
Survival distribution of the entire HCC cohort (*n* = 3690), stratified by renal insufficiency (RI, eGFR <60 mL/min/1.73 m^2^) using the Kaplan–Meier method with log-rank test. Patients with renal insufficiency had significantly decreased long-term survival compared to those without renal insufficiency (*p* < 0.001).

The survival distributions of patients with and without RI are illustrated in Figure 2. During a mean follow-up period of 37 (median, 31) months, patients with RI had significantly decreased survival compared to patients without RI (*p* < 0.001, Figure 2). The median survival in patients with RI and without RI was 22 months (95% CI: 18–26 months) and 36 months (95% CI: 32.5–39.5 months), respectively. The one- and three-year survival rates of patients with RI and without RI were 60% versus 69%, and 39% versus 50%, respectively. Among patients with RI, 730 (73%) patients died during the study period. The cause of death can be divided into liver-related and non-liver related etiology. In total, 407 (56%) patients died of liver-related causes such as tumor progression and hepatic failure, 107 (14%) patients died of direct renal failure and 216 (30%) patients died of non-liver related causes including sepsis and others.

Among the 1000 patients with RI, there were 36 (3.6%) patients undergoing dialysis at the time of diagnosis. The median survival was 16 (95% CI: 9–23) months in patients (*n* = 36) with dialysis and 25 (95% CI: 21–29) months in patients without dialysis (*n* = 964; *p* = 0.05).

In univariate analysis, older age, male, HBsAg seropositivity, low serum albumin, high serum bilirubin, high serum alanine aminotransferase (ALT), thrombocytopenia, prolonged prothrombin time (PT), RI, higher AFP level, multiple tumors, larger tumor size, presence of vascular invasion, distant metastasis, presence of ascites, poor performance status, and ALBI grade 2 and 3 were all associated with decreased long-term survival. In the multivariate Cox model, serum albumin and bilirubin levels were not included because of high collinearity with ALBI grade. Independent prognostic predictors included age older than 65 years (HR: 1.160, *p* < 0.001), male (HR: 1.207, *p* < 0.001), eGFR <60 mL/min/1.73 m^2^ (HR: 1.234, *p* < 0.001), AFP ≥20 ng/mL (HR: 1.856, *p* < 0.001), multiple tumors (HR: 1.136, *p* = 0.003), tumor size >3 cm (HR: 1.793, *p* < 0.001), vascular invasion (HR: 2.486, *p* < 0.001), distant metastasis (HR: 1.494, *p* < 0.001), performance status 1–2 (HR: 1.515, *p* < 0.001), performance status 3–4 (HR: 2.511, *p* < 0.001), ALBI grade 2 (HR: 1.855, *p* < 0.001), and ALBI grade 3 (HR: 2.916, *p* < 0.001), which were significantly linked with decreased survival (Table 2).

### 3.3. Survival of Patients with and without RI Based on ALBI Grade

**Figure 3:**
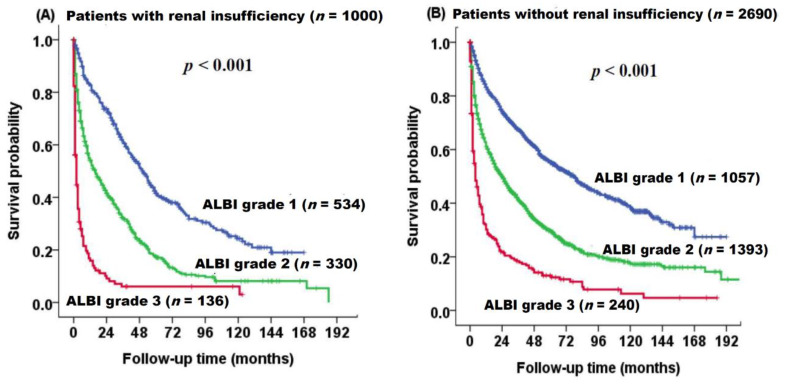
Survival distribution of HCC patients with (**A**) and without (**B**) renal insufficiency based on ALBI grade analyzed by the Kaplan–Meier method with log-rank test. Patients with ALBI grade 2 and 3 had increased risks of mortality compared to those with ALBI grade 1 in both patient cohorts (*p* < 0.001).

The survival distributions of patients with and without RI based on ALBI grade are shown in Figure 3. The ALBI grade can stratify three different prognostic groups in patients with RI (*p* < 0.001, Figure 3A) and without RI (*p* < 0.001, Figure 3B).

### 3.4. Prognostic Predictors in Patients with RI

In univariate analysis, serum HBsAg seropositivity, low serum albumin level, high serum bilirubin level, high serum ALT level, thrombocytopenia, PT, higher serum AFP, multiple tumors, larger tumor size, presence of vascular invasion, distant metastasis, presence of ascites, poor performance status, and ALBI grade 2 and ALBI grade 3 were linked with decreased survival in HCC patients with RI. The multivariate Cox analysis revealed serum AFP ≥20 ng/mL (HR: 1.727, *p* < 0.001), tumor size >3 cm (HR: 1.850, *p* < 0.001), presence of vascular invasion (HR: 1.809, *p* < 0.001), distant metastasis (HR: 1.494, *p* < 0.001), presence of ascites (HR: 1.288, *p* = 0.043), performance status 1–2 (HR: 1.413, *p* < 0.001), performance status 3–4 (HR: 1.985, *p* < 0.001), ALBI grade 2 (HR: 1.695, *p* < 0.001), and ALBI grade 3 (HR: 2.878, *p* < 0.001) as independent prognostic predictors of poor survival (Table 3).

In subgroup analysis of patients with RI undergoing curative treatment, age older than 65 years (HR: 1.385, *p* = 0.026), serum AFP ≥20 ng/mL (HR: 1.697, *p* < 0.001), tumor size >3 cm (HR: 1.553, *p* < 0.001), vascular invasion (HR: 2.321, *p* < 0.001), distant metastasis (HR: 2.335, *p* < 0.001), performance status 1–2 (HR: 1.488, *p* < 0.001), performance status 3–4 (HR: 1.958, *p* < 0.001), ALBI grade 2 (HR: 1.682, *p* < 0.001), and ALBI grade 3 (HR: 7.115, *p* < 0.001) were associated with poor survival in multivariate analysis (Table 4). Among those with RI undergoing non-curative treatment, serum AFP ≥20 ng/mL (HR: 1.648, *p* < 0.001), multiple tumors (HR: 1.217, *p* < 0.001), tumor size >3 cm (HR: 1.914, *p* < 0.001), vascular invasion (HR: 1.662, *p* < 0.001), distant metastasis (HR: 1.395, *p* = 0.021), presence of ascites (HR: 1.249, *p* = 0.033), performance status 1–2 (HR: 1.314, *p* = 0.025), performance status 3–4 (HR: 1.876, *p* < 0.001), ALBI grade 2 (HR: 1.659, *p* < 0.001), and ALBI grade 3 (HR: 2.081, *p* < 0.001) were associated with poor survival in the Cox model (Table 5). Of the patients receiving best supportive care, the median survival was four (95% CI: 2.7–5.2) months in patients with RI and eight (95% CI: 5.0–11.0) months in patients without RI (*p* < 0.001).

## 4. Discussion

Patients with HCC often have liver cirrhosis which may further predispose to RI [26,27]. In this study, more than one-quarter (27%) of HCC patients had RI; in addition, 27% of patients had advanced cirrhosis (CTP class B or C). Patients with RI are characterized by older age, female predominance, higher prevalence of DM, larger tumor volume, advanced cirrhosis, and poor performance status, indicating that RI is an indicator of advanced cancer stage and poor general condition. These unfavorable factors may result in a lower chance to undergo aggressive treatment. Notably, in this 11,070 persons/year study, we show that patients with RI had a 25% increased risk of mortality compared to patients without RI. Consistent with previous studies [7,12,28], our results clearly demonstrate that RI is a negative prognostic predictor in HCC, and suggest that incorporation of renal function into the prognostic model for HCC may provide additional crucial clinical information.

The severity of liver cirrhosis is a major predictor for HCC [19]. The CTP classification is widely used to assess the severity of liver cirrhosis, but its predictive power is limited. Alternatively, the MELD score is more often used in the setting of cirrhotic patients awaiting liver transplantation [29,30]. An intrinsic deficiency for these two systems is that about 20% of HCC patients had normal or only slightly decreased liver functional reserve at the time of diagnosis [31]. Therefore, the predictive accuracy of CTP classification and MELD score in HCC patients with less severe liver injury has been greatly challenged.

ALBI grade is a more recently introduced marker to evaluate liver function in HCC [13,14,15,16]. In comparison with CTP and MELD, the ALBI grade is based solely on two subjective variables (serum albumin and serum bilirubin) which can be readily obtained from routine blood tests. Given this advantage, the predictive accuracy of ALBI grade in HCC patients with RI has not been evaluated. Our results show that ALBI grade can discriminate overall survival in HCC patients with and without RI, suggesting that ALBI grade is an important prognostic predictor for these patients [13,15,16]. Notably, for HCC patients with RI, ALBI grade 2 and 3 patients had significantly increased risk of mortality compared with those with ALBI grade 1 in both curative and non-curative treatment groups, suggesting ALBI grade is a consistent and feasible marker in prognostic prediction for HCC patients with RI.

In addition to liver functional reserve, tumor burden and performance status are also important factors in management HCC. Consistent with previous findings [32,33], our data indicate that higher tumor burden (larger tumor size, multiple tumors, vascular invasion, distant metastasis, and high serum AFP level) and ascites were associated with decreased survival in patients with RI. Ascites is known as an indicator for portal hypertension. In addition, the presence of ascites was reported to be associated with increased risk of mortality in HCC [34,35]. Moreover, consistent with our earlier study [20], patients with a poor performance status had 41% to 98% increased risk of mortality among those with RI. Taken together, ALBI grade, tumor burden, and performance status are all crucial prognostic factors in HCC patients with RI.

In this study, only a small proportion (3.6%) of RI patients received dialysis. Consistently, we found that patients without dialysis survived longer than patients with dialysis [36]. However, the impact of dialysis requires further large-scale studies to confirm.

In patients with RI, ALBI grade 3 was associated with the worst prognosis. In this group, 97 (72%) patients received best supportive care, 16 (12%) patients received transarterial chemoembolization, 8 (6%) patients received local ablation therapy, 7 (5%) patients received chemotherapy, 4 (3%) patients received targeted therapy, 2 (2%) patients received surgical resection, and 2 (2%) patients received liver transplantation.

There are a few limitations in this study. Firstly, this study was performed in an area with high prevalence of HBV infection which is different from Western countries and Japan where HCV infection is the major risk factor for HCC. Secondly, the etiology of RI was not specifically determined in this study, and some patients may have other intrinsic renal parenchymal disease. Thirdly, this is a single-center retrospective study and further study is needed to validate our results.

## 5. Conclusions

HCC patients with RI have decreased overall survival compared to those without RI. The ALBI grade can discriminate the survival among patients with RI independent of treatment strategy and is a feasible and reliable prognostic tool to assess the severity of liver dysfunction in this special patient population.

## Figures and Tables

**Table 1:** Comparison of baseline characteristics of hepatocellular carcinoma (HCC) patients with and without renal insufficiency (RI).

Variables		All Patients (*n* = 3690)	Patients with RI (*n* = 1000)	Patients without RI (*n* = 2690)	*p*-Value
Age (years, mean ± SD)		65 ± 13	72 ± 11	62 ± 13	<0.001
Male/Female, *n* (%)		2815/875 (76/24)	732/268 (73/27)	2083/83 (77/23)	0.008
Etiologies of liver disease					<0.001
	HBV, *n* (%)	1475 (40)	308 (31)	1165 (43)	
	HCV, *n* (%)	809 (22)	249 (25)	560 (21)	
	HBV + HCV, (%)	133 (4)	35 (4)	98 (4)	
	Others, (%)	1275 (35)	408 (40)	867 (32)	
Laboratory values (mean ± SD)					
	Albumin (g/L)	3.6 ± 0.6	3.5 ± 0.7	3.7 ± 0.6	0.001
	Bilirubin (mg/dL)	1.5 ± 2.9	1.7 ± 3.9	1.5 ± 2.4	0.003
	ALT (IU/L)	69 ± 88	63 ± 94	72 ± 85	0.043
	AST (IU/L)	98 ± 207	113 ± 345	94 ± 120	0.399
	Creatinine (mg/dL)	1.2 ± 1.1	2.0 ± 1.9	0.9 ± 0.2	<0.001
	Sodium (mmol/L)	138 ± 5	137 ± 6	139 ± 4	0.001
	INR of PT	1.1 ± 0.2	1.1 ± 0.3	1.1 ± 0.1	0.615
	Platelet (1000 uL/L)	172 ± 110	170 ± 101	173 ± 113	0.371
	AFP (ng/mL) median (IQR)	44 (8–794)	31 (6–886)	51 (9–764)	0.004
	Tumor nodules (single/multiple), *n* (%)	2354/1336 (64/36)	653/347 (65/35)	1701/989 (63/37)	0.248
	Tumor size, Mean ± SD	6.03 ± 4.5	6.22 ± 4.5	5.97 ± 4.5	0.766
	Tumor size >3 cm, *n* (%)	2407 (65)	681 (68)	1726 (64)	0.026
TTV median, (IQR) Mean ± SD		47 (9–381)	65 (10–609) 391 ± 844	47 (9–381) 372 ± 732	0.382
Vascular invasion, *n* (%)		897 (24)	227 (23)	670 (25)	0.167
Distant metastasis, *n* (%)		355 (10)	103 (10)	252 (9)	0.414
Ascites, *n* (%)		837 (23)	261 (26)	576 (21)	0.003
DM, *n* (%)		936 (26)	360 (36)	576 (21)	<0.001
CTP class (A/B/C), *n* (%)		2706/812/172 (73/22/5)	718/226/56 (72/22/6)	2169/451/70 (80/17/3)	<0.001
CTP score (mean ± SD)		6.1 ± 1.5	6.4 ± 1.8	6.0 ± 1.4	0.254
ALBI grade					<0.001
	1	1387 (38)	330 (33)	664 (36)	
	2	1927 (52)	534 (53)	996 (54)	
	3	376 (10)	136 (14)	184 (10)	
Performance status					<0.001
	0	2168 (60)	514 (51)	1654 (62)	
	1	770 (21)	167 (17)	603 (32)	
	2	399 (11)	151 (15)	248 (9)	
	3–4	353 (10)	168 (17)	185 (7)	
BCLC, *n* (%)					<0.001
	0	295 (8)	65 (7)	230 (9)	
	A	908 (24)	216 (22)	692(26)	
	B	610 (17)	157 (15)	453 (17)	
	C	1459 (40)	371 (37)	1088 (40)	
	D	418 (11)	191 (19)	227 (8)	
Treatment					<0.001
	Surgical resection	1069 (29)	231 (23)	838 (31)	
	Liver transplantation	18 (1)	4 (1)	14 (1)	
	Percutaneous ablation	672(18)	213 (21)	459 (17)	
	TACE	1047 (28)	292 (29)	755 (28)	
	Targeted therapy	123 (3)	26 (3)	97 (4)	
	Chemotherapy	174 (5)	38 (4)	136 (5)	
	Best supportive care	587 (16)	196 (19)	391 (15)	

Targeted therapy, chemotherapy, and best supportive care were defined as ‘Others’ treatments. Abbreviations: ALBI, albumin-bilirubin; ALT, alanine aminotransferase; AST, aspartate aminotransferase; AFP, α-fetoprotein; BCLC, Barcelona Clinic Liver Cancer; CTP, Child–Turcotte–Pugh; DM, diabetes mellitus; HBV, hepatitis B virus; HCV, hepatitis C virus; INR of PT, international normalized ration of prothrombin time; IQR, interquartile range; IU, International Unit; MELD, model of end-stage liver disease; SD, standard deviation; TACE, transarterial chemoembolization; TTV, total tumor volume.

**Table 2:** Univariate and multivariate survival analysis of all study patients (*n* = 3690).

Overall Survival		Number	Univariate Analysis			Multivariate Analysis		
			HR	CI	*p*-Value	HR	CI	*p*-Value
Age (≤65/>65 years)		1839/1851	1.200	1.107–1.300	<0.001	1.160	1.064–1.264	<0.001
Sex (male/female)		2815/875	1.177	1.069–1.296	0.001	1.207	1.095–1.332	<0.001
HBsAg (negative/positive)		1703/1987	1.108	1.023–1.210	0.012			
Anti-HCV (negative/positive)		2579/1111	0.964	0.884–1.051	0.407			
Albumin level (≥3.5/<3.5 g/dL)		2403/1287	2.315	2.133–2.513	<0.001	1.670	1.522–1.832	<0.001
Bilirubin level (≤1.5/>1.5 mg/dL)		2899/791	2.048	1.867–2.247	<0.001	1.433	1.293–1.588	<0.001
ALT (≤40/>40 IU/L)		1555/2135	1.235	1.138–1.340	<0.001			
Platelet (≥150,000/<150,000 μL)		1903/1787	1.195	1.104–1.295	<0.001			
INR of PT (<1.1/≥1.1)		2422/1267	1.653	1.521–1.796	<0.001			
eGFR (≥60/<60 mL/min/1.73 m^2^)		2690/1000	1.414	1.296–1.549	<0.001	1.234	1.123–1.355	<0.001
AFP (<20/≥20 ng/mL)		1494/2196	2.020	1.855–2.199	<0.001	1.856	1.702–2.025	<0.001
Multiple tumors (no/yes)		2354/1336	1.318	1.215–1.430	<0.001	1.136	1.045–1.234	0.003
Tumor size (≤3/>3 cm)		1283/2407	2.234	2.043–2.442	<0.001	1.793	1.632–1.970	<0.001
Vascular invasion (no/yes)		2793/897	4.296	3.925–4.703	<0.001	2.486	2.240–2.759	<0.001
Distant metastasis (no/yes)		3335/355	3.534	3.124–3.998	<0.001	1.494	1.309–1.705	<0.001
Ascites (no/yes)		2853/837	1.653	1.507–1.813	<0.001			
Performance status								
	0	2168	1			1		
	1–2	1169	2.139	1.957–2.337	<0.001	1.515	1.380–1.663	<0.001
	3–4	353	5.365	4.728–6.087	<0.001	2.511	2.172–2.904	<0.001
ALBI grade								
	Grade 1	1387	1			1		
	Grade 2	1927	2.074	1.894–2.272	<0.001	1.855	1.689–2.036	<0.001
	Grade 3	376	4.675	4.086–5.348	<0.001	2.916	2.513–3.383	<0.001

Abbreviations: AFP, α-fetoprotein; ALBI, albumin-bilirubin; ALT, alanine aminotransferase; anti-HCV, hepatitis C virus antibody; CI, confidence interval; eGFR, estimated glomerular filtration rate; HBsAg, hepatitis B virus surface antigen; HR, hazard ratio; INR of PT, international normalized ration of prothrombin time.

**Table 3:** Univariate and multivariate survival analysis in HCC patients with renal insufficiency (*n* = 1000).

Overall Survival		Number	Univariate Analysis			Multivariate Analysis		
			HR	CI	*p-*Value	HR	CI	*p-*Value
Age (≤65/>65 years)		256/744	1.120	0.945–1.326	0.192			
Sex (male/female)		732/268	0.933	0.789–1.103	0.418			
HBsAg (negative/positive)		589/411	1.197	1.031–1.390	0.018			
Anti-HCV (negative/positive)		669/331	0.957	0.820–1.117	0.576			
Albumin level (≥3.5/<3.5 g/dL)		581/419	2.286	1.970–2.652	<0.001	1.579	1.338–1.864	<0.001
Bilirubin level (≤1.5/>1.5 mg/dL)		796/204	2.455	2.063–2.9222	<0.001	1.536	1.266–1.863	<0.001
ALT (≤40/>40 IU/L)		485/515	1.297	1.121–1.502	<0.001			
Platelet (≥150,000/<150,000 μL)		503/497	1.187	1.026–1.373	0.021			
INR of PT (<1.1/≥1.1)		663/337	1.700	1.459–1.980	<0.001			
AFP (<20/≥20 ng/mL)		443/557	1.971	1.695–2.292	<0.001	1.727	1.481–2.013	<0.001
Multiple tumors (no/yes)		653/347	1.410	1.214–1.637	<0.001			
Tumor size (≤3/>3 cm)		319/681	2.293	1.942–2.707	<0.001	1.850	1.551–2.207	<0.001
Vascular invasion (no/yes)		773/227	3.622	3.053–4.297	<0.001	1.809	1.491–2.195	<0.001
Distant metastasis (no/yes)		897/103	3.343	2.671–4.185	<0.001	1.494	1.175–1.900	0.001
Ascites (no/yes)		739/261	1.924	1.638–2.261	<0.001	1.228	1.036–1.456	0.043
Performance status								
	0	514	1			1		
	1–2	318	2.059	1.739–2.437	<0.001	1.413	1.180–1.693	<0.001
	3–4	168	4.574	3.755–5.571	<0.001	1.985	1.553–2.536	<0.001
ALBI grade								
	Grade 1	330	1			1		
	Grade 2	534	2.107	1.776–2.499	<0.001	1.695	1.418–2.026	<0.001
	Grade 3	136	5.662	4.468–7.175	<0.001	2.878	1.972–3.444	<0.001

Abbreviations: AFP, α-fetoprotein; ALBI, albumin-bilirubin; ALT, alanine aminotransferase; anti-HCV, antibody against hepatitis C virus; HBsAg, hepatitis B virus surface antigen; INR of PT, international normalized ratio of prothrombin time.

**Table 4:** Univariate and multivariate survival analysis in HCC patients with renal insufficiency undergoing curative treatment (*n* = 448).

Overall Survival		Number	Univariate Analysis			Multivariate Analysis		
			HR	CI	*p-*Value	HR	CI	*p-*Value
Age (≤65/>65 years)		114/334	1.338	1.018–1.757	0.037	1.385	1.041–1.844	0.026
Sex (male/female)		335/113	0.908	0.695–1.186	0.479			
HBsAg (negative/positive)		262/186	1.316	1.037–1.671	0.024			
Anti-HCV (negative/positive)		295/153	0.946	0.744–1.203	0.650			
Albumin level (≥3.5/<3.5 g/dL)		294/154	2.453	1.931–3.115	<0.001	1.748	1.340–2.281	<0.001
Bilirubin level (≤1.5/>1.5 mg/dL)		380/68	2.522	1.876–3.391	<0.001	1.950	1.389–2.736	<0.001
ALT (≤40/>40 IU/L)		227/221	1.311	1.040–1.653	0.014			
Platelet (≥150,000/<150,000 μL)		207/241	0.992	0.786–1.250	0.943			
INR of PT (<1.1/≥1.1)		311/137	1.935	1.519–2.466	<0.001			
AFP (<20/≥20 ng/mL)		239/209	1.733	1.375–2.184	<0.001	1.697	1.333–2.082	<0.001
Multiple tumors (no/yes)		342/106	0.965	0.739–1.260	0.795			
Tumor size (≤3/>3 cm)		191/257	1.838	1.450–2.331	<0.001	1.553	1.208–1.996	<0.001
Vascular invasion (no/yes)		390/58	3.948	2.896–5.383	<0.001	2.321	1.639–3.287	<0.001
Distant metastasis (no/yes)		421/27	4.340	2.819–6.683	<0.001	2.335	1.476–1.695	<0.001
Ascites (no/yes)		395/53	0.716	0.509–1.008	0.056			
Performance status								
	0	281	1			1		
	1–2	122	1.832	1.405–2.389	<0.001	1.488	1.128–1.962	0.005
	3–4	45	4.808	3.380–6.840	<0.001	1.958	1.259–3.045	<0.001
ALBI grade								
	Grade 1	185	1			1		
	Grade 2	220	2.036	1.577–2.629	<0.001	1.682	1.293–2.189	<0.001
	Grade 3	43	10.676	7.203–15.825	<0.001	7.115	4.395–11.520	<0.001

Abbreviations: AFP, α-fetoprotein; ALBI, albumin-bilirubin; ALT, alanine aminotransferase; anti-HCV, antibody against hepatitis C virus; HBsAg, hepatitis B virus surface antigen; INR of PT, international normalized ratio of prothrombin time.

**Table 5:** Univariate and multivariate survival analysis in HCC patients with renal insufficiency undergoing non-curative treatment (*n* = 552).

Overall Survival		Number	Univariate Analysis			Multivariate Analysis		
			HR	CI	*p*	HR	CI	*p*
Age (≤65/>65 years)		142/410	0.956	0.770–1.187	0.683			
Sex (male/female)		397/155	1.282	1.033–1.590	0.024			
HBsAg (negative/positive)		327/255	0.889	0.734–1.077	0.231			
Anti–HCV (negative/positive)		374/178	0.903	0.738–1.105	0.320			
Albumin level (≥3.5/<3.5 g/dL)		287/265	1.931	1.596–2.335	<0.001	1.459	1.102–1.932	0.008
Bilirubin level (≤1.5/>1.5 mg/dL)		416/136	2.158	1.739–2.679	<0.001	1.401	1.134–1.730	0.002
ALT (≤40/>40 IU/L)		258/294	1.266	1.048–1.529	0.014			
Platelet (≥150,000/<150,000 μL)		296/256	1.246	1.032–1.505	0.022			
INR of PT (<1.1/≥1.1)		352/200	1.488	1.223–1.811	<0.001			
AFP (<20/≥20 ng/mL)		204/348	1.905	1.556–2.333	<0.001	1.648	1.342–2.025	<0.001
Multiple tumors (no/yes)		311/241	1.371	1.136–1.655	<0.001			
Tumor size (≤3/>3 cm)		128/424	2.639	1.858–3.021	<0.001	1.914	1.470–2.491	<0.001
Vascular invasion (no/yes)		383/169	2.896	2.353–3.563	<0.001	1.662	1.317–2.097	<0.001
Distant metastasis (no/yes)		476/76	2.621	2.013–3.413	<0.001	1.395	1.052–1.849	<0.021
Ascites (no/yes)		344/208	1.679	1.385–2.037	<0.001			
Performance status								
	0	233						
	1–2	196	1.965	1.572–2.457	<0.001	1.314	1.035–1.699	0.025
	3–4	123	3.665	2.866–4.688	<0.001	1.876	1.395–2.521	<0.001
ALBI grade								
	Grade 1	145						
	Grade 2	314	1.933	1.532–2.437	<0.001	1.659	1.297–2.122	<0.001
	Grade 3	93	3.531	2.610–4.778	<0.001	2.081	1.466–2.952	<0.001

Abbreviations: AFP, α-fetoprotein; ALBI, albumin-bilirubin; ALT, alanine aminotransferase; anti-HCV, antibody against hepatitis C virus; HBsAg, hepatitis B virus surface antigen; INR of PT, international normalized ratio of prothrombin time.

## Abbreviations

ALBI	albumin-bilirubin
ALT	alanine aminotransferase
Anti-HCV	antibody for hepatitis C virus
AST	aspartate aminotransferase
AFP	α-fetoprotein
BCLC	Barcelona Clinic Liver Cancer
CTP	Child–Turcotte–Pugh
eGFR	estimated glomerular filtration rate
DM	diabetes mellitus
HBsAg	hepatitis B surface antigen
HBV	hepatitis B virus
HCV	hepatitis C virus
HR	hazard ratio
INR of PT	international normalized ratio of prothrombin time
MDRD	modification of diet in renal disease
MELD	model of end-stage liver disease
TACE	transarterial chemoembolization
RI	renal insufficiency
SD	standard deviation
